# Modelling Sensory Limitation: The Role of Tree Selection, Memory and Information Transfer in Bats’ Roost Searching Strategies

**DOI:** 10.1371/journal.pone.0044897

**Published:** 2012-09-13

**Authors:** Ireneusz Ruczyński, Kamil A. Bartoń

**Affiliations:** 1 Mammal Research Institute PAS, Białowieża, Poland; 2 Field Station, University of Würzburg, Fabrikschleichach, Rauhenebrach, Germany; Arizona State University, United States of America

## Abstract

Sensory limitation plays an important role in the evolution of animal behaviour. Animals have to find objects of interest (e.g. food, shelters, predators). When sensory abilities are strongly limited, animals adjust their behaviour to maximize chances for success. Bats are nocturnal, live in complex environments, are capable of flight and must confront numerous perceptual challenges (e.g. limited sensory range, interfering clutter echoes). This makes them an excellent model for studying the role of compensating behaviours to decrease costs of finding resources. Cavity roosting bats are especially interesting because the availability of tree cavities is often limited, and their quality is vital for bats during the breeding season. From a bat’s sensory point of view, cavities are difficult to detect and finding them requires time and energy. However, tree cavities are also long lasting, allowing information transfer among conspecifics. Here, we use a simple simulation model to explore the benefits of tree selection, memory and eavesdropping (compensation behaviours) to searches for tree cavities by bats with short and long perception range. Our model suggests that memory and correct discrimination of tree suitability are the basic strategies decreasing the cost of roost finding, whereas perceptual range plays a minor role in this process. Additionally, eavesdropping constitutes a buffer that reduces the costs of finding new resources (such as roosts), especially when they occur in low density. We conclude that natural selection may promote different strategies of roost finding in relation to habitat conditions and cognitive skills of animals.

## Introduction

All animals face the problem of finding resources, such as food and shelters that are limited and maybe difficult to find [1]–[4]. An efficient sensory system is extremely important in this context, because it allows for gathering crucial information for the detection of suitable resources. However, there is a trade-off between the energetic cost of maintaining a sensory structure encoding a particular sensory modality and the amount of reliable, germane information obtained [5]. Consequently, the possibilities for improving senses (such as increasing the range for detection) in the course of evolution are limited, and selection pressures may favour other physiological, morphological or behavioural adaptations. Here we consider three alternative strategies for finding resources: 1) selecting places or objects that offer the highest chance of success [6], [7]; 2) memorising the resource distribution once discovered [7], [8] or; 3) obtaining information from conspecifics [9], [10].

We hypothesize that the effectiveness of such compensatory strategies is substantial, but it depends on environmental and sensory limitations, as well as abilities to remotely evaluate suitability of the resources. The importance of the compensating strategy should increase with stronger limitation. The search of forest dwelling bats for tree roosts is a perfect model system for testing these hypotheses, as tree roosts are important for bats, relatively stable in their availability over time and space and energetically costly to find [4], [11], [12]. During the last decades, knowledge of sensory limitation, associative learning, memory and information transfer in bats has accumulated (e.g. [13]–[16]). We are therefore now able to integrate theory with empirical field and laboratory data. In the current study we do just that, and use simulations in order to identify the benefits of different strategies for finding new resources, such as tree roosts.

### Importance of Roost Trees for Bats and Roosting Behaviour

Over half of the more than 1200 species of bats use tree cavities during at least part of the year [17]. Suitable roosts have to fulfil many conditions, such as offering protection from unfavourable weather and predators, or providing a suitable microclimate for temperature regulation [18]–[20], reviewed in [17]. Tree dwelling bats do not excavate, thus they are fully dependent on the presence of suitable tree cavities (see [17] for a review). Bats use holes created by woodpeckers, cavities left by broken-off branches or spaces beneath bark (hereafter referred to as “tree cavities” or “roosts” if used by bats). Roosts are especially important during the reproductive period, because their quality may influence the development of juveniles or breeding success (e.g. [21], [22], reviewed in [17]). Most tree dwelling species change roosts every few days and return to them later; this further increases the necessary pool of suitable tree cavities [12], [23]. Bat social groups are often fission-fusion societies, which spread over multiple tree cavities, with the number of bats in each tree ranging from a few to several hundred individuals (reviewed in [17]). Consequently, when changing roosts bats need knowledge about the local distribution of tree cavities, as well as an efficient system of information transfer and decision making [24], [25].

**Figure 1:**
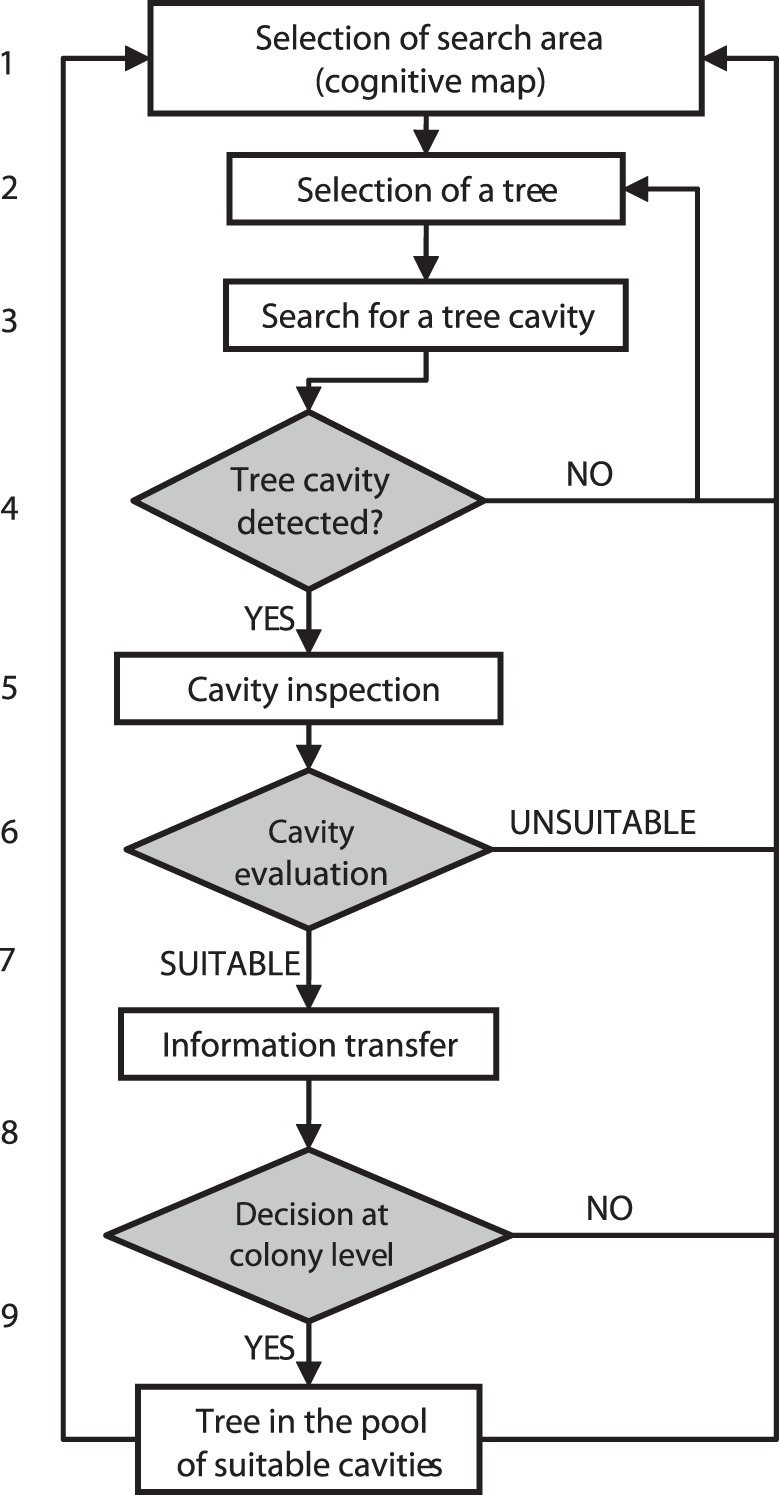
Process of finding new tree cavities by bats and acquisition of information about the presence and distribution of tree cavities in the forest.

### Finding Tree Cavities

#### Echolocation and visual limitations

Bats use echolocation for orientation in space, as well as for detecting, identifying and localizing food [26]. The detection range for an object does not exceed 90 m, and often it is limited to a few meters [26], [27]. Vision, in contrast to echolocation, is a passive modality that allows for long distance orientation, which is important for building large-scale “cognitive maps” essential to navigation [26], [28], [29]). Bats can use vision to localize objects or food (e.g. [30]–[32]. Use of vision, however, is also strongly limited by object size and light intensity [33]. Experiments conducted under laboratory conditions suggest that visual cues may lead to higher detection of cavity entrances but the effect is limited only to specific conditions [4], [34], [35].

#### Environmental limitations

The period of cavity creation may take many years, which is evidenced by the fact that more tree cavities are observed in older trees. The best predictors for tree cavity occurrence are tree diameter and amount of dead wood in the canopy [36], [37]. Woodpeckers create cavities in trees where growth has been suppressed internally for many years and that show signs of moderate to high crown dieback [38]. Such trees are usually larger and taller with broken or dead branches, and may lack bark or even consist only of the trunk. Most tree dwelling bats select such trees (for review, see [39], but it is not clear whether this is because of cavity features or the high chances for finding cavities (or both). Trees selected by bats nonetheless have features that due to size and texture could be detectable by both vision and/or echolocation [40].

The proportion of trees containing suitable cavities is poorly known. Sedgeley & O’Donnell [23] determined that in well-preserved forests only 1.3% of trees contained cavities suitable as roosts. Nonetheless, bats were able to find new roosts almost every day, rarely reusing the cavities [23]. In managed forests, where younger trees dominate, availability of suitable cavities is clearly lower and the benefits of using compensation strategies to find roosts efficiently are expected to be higher.

**Figure 2:**
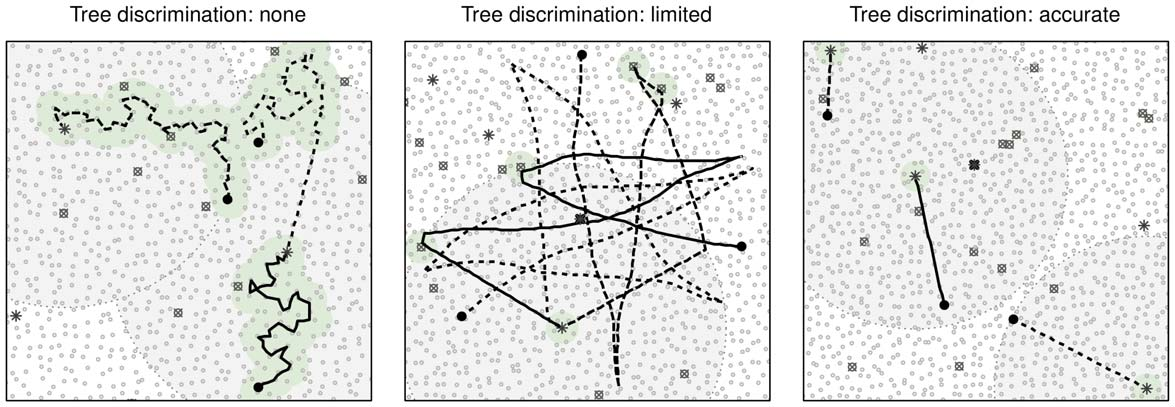
Examples of simulated trajectories of bats searching for roosts, for each of three discrimination abilities. Lines represent trajectories of three bats distinguished with different line patterns. Starting points are denoted with a black bullet; small gray circles represent unsuitable trees (type A), crossed circles – confounding trees (type B), stars – suitable trees (with cavity, type C). Large shaded circles illustrate a calling range, with the bat calling from the tree in the centre of the circle, small shaded circles around visited trees show the range within which the tree can be detected. Within both of these perceptual ranges bats fly directly to the target tree. Bats that have wandered beyond the ‘searching range’, will reorient towards the current roost (most visible in the middle panel). In the right hand panel the simulation assumes that the bats follow a ‘memorized’ mental map and go directly to the closest suitable tree. Note that for clarity the proportions in the figure differ from those used in actual simulations.

**Table 1:** Parameters used in the model and their values. See methods section for details (1 second  = 1 time step, 1 m  = 0.04 spatial units/1 spatial unit = 25 m).

Parameter	Values
Average distance between trees (*d_tree_*)	10 m
Step length (*l*)	5 m
Flight speed	5 m/s
Range of uniform random variation in the turning angles (−θ, +θ)	−0.2 π = 11.5°
Uncertainty of tree distance assessment (±range of uniform randomvariation in the distance to the tree, perceived by flying bat)	7.5 m
Searching range (*r_search_*)	500 m
Looping probability (per-step probability of a random turn whileoutside of searching range)	0.02
Tree cavity search time (*t_insp_*)	420 s
Perceptual range (*r_p_*)	5 m, 90 m, infinite^1^
Call detectability range (*r_call_*)	150 m
Group size (*n*)	1, 5, 10, 75, 100 individuals
Tree proportion (type A/type B/type C)	990/0/10, 999/0/1, 990/9/1, 900/99/1

1) Note that since the individual responds only to the nearest recognized tree (of type depending on the discrimination level, see ‘Methods’), the effective ‘infinity’ is achieved by setting a perceptual range larger than the largest nearest neighbour distance between the recognized trees.

### Compensation Strategies

#### Selection and associative learning

Bats are able to associate shapes with the presence of food, reverse-evaluate cues, generalize shapes and finally select objects of interest [14], [41]–[44]. Many studies have demonstrated their developed learning abilities, such as associative learning and even learning by observation [14]; even plant classification from bat-like echolocation signals is possible [45]–[47]. This, together with the preference for specific tree parameters indicating a higher probability for finding cavities, suggests that bats may rely on experience when selecting trees (e.g. [39] for review [17]). In the context of strong sensory limitation, selection of high-quality trees (which we here call ‘tree discrimination’) may be the most plausible strategy to decrease the costs of having to find new tree cavities.

#### Memory

Bats are long lived animals [48], thus they can gain benefits from learning and memory retention throughout their life. The memory of bats is specifically protected during hibernation, which indicates its importance for these animals [35]. Once a roost is found, it can be used for many years [11], [49]. Reuse of known roosts minimizes costs of resource discovery. Memory can also play an important role in decision making processes: where and when to search for new cavities. During hunting, bats are able to build “cognitive maps” for orientation in space [29], and therefore to gather information about trees that could contain suitable cavities.

#### Eavesdropping as information transfer

Bats are gregarious and a few to hundreds of individuals may roost in a single cavity (for review, see [12], [17]). Through grouping, they benefit from better protection from predators, increased movement efficiency, improved food discovery etc. [2], [50]. Roosts are also recognised as “information centres” [51]. Bats can transfer information about the presence of cavities to conspecifics either actively or passively, using acoustic calls or behaviours such as swarming or following [1], [13], [52]–[55]. Eavesdropping on calls of bats in roosts is the simplest way of gathering information about new cavities [13], [53], [56]. The potential role of group size in decreasing the costs of finding new cavities is poorly understood in the context of eavesdropping.

The aims of our study were: 1) to estimate and compare the benefits connected with each compensation strategy (tree discrimination, information transfer, and memory) in situations of strong and weak sensory limitation (i.e. small and large perceptual range), and low and high tree cavity availability; 2) to estimate the benefits associated with tree selection (discrimination) and information transfer when the effectiveness to select suitable trees is high or low; 3) to estimate the efficiency of information transfer in groups of different size.

**Figure 3:**
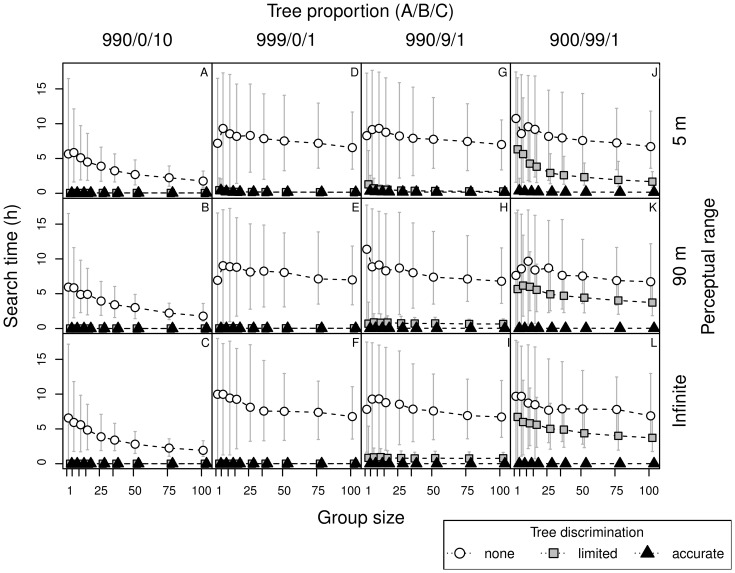
Roost search times by bats, in relation to perceptual range, group size, tree discrimination skills and forest type. Search time of new cavities by bats with short (5 m) and long (90 m) perceptual range, and knowledge about distribution of trees in the forest (infinite perceptual range) in relation to tree discrimination skills (none, limited, accurate), group size (1, 5, 10, 75,100) and proportion of tree types in the forest: unsuitable, without cavities (type A), confounding (apparently suitable but without cavities, type B), and with a cavity (type C), denoted as ‘A/B/C’.

**Table 2:** Mean search time (±SD) of tree cavities by bats in relation to forest type, bats’ perceptual range and skills of tree discrimination.

Discrimination of trees by bats	Forest type											
	High density of trees with cavities			Low density of trees with cavities								
	990/0/10			999/0/1			990/9/1			900/99/1		
	Perceptual range			Perceptual range			Perceptual range			Perceptual range		
	5m	90m	infinity	5m	90m	infinity	5m	90m	infinity	5m	90m	infinity
none	232.7±89.9	239.7±90.6	248±100.9	470.9±50.2	481±49.8	506.7±73.4	492.5±46.3	504.4±82.9	485.3±57.1	504.7±74.7	478.3±56.42	497.9±56.27
limited	2.9±0.13	0.2±0.0	0.2±0.0	13.3±5.4	1.9±0.3	0.5±0.0	31.2±19.2	45.1±5.0	49.6±4.0	209.7±98.5	301.3±52.32	307.6±59.33
accurate	2.9±0.2	0.2±0.0	0.2±0.0	13.1±5.09	2.0±0.13	0.5±0.01	12.4±3.22	1.9±0.2	0.5±0.0	12.7±3.9	2.0±0.23	0.5±0.01

Proportion of trees in forests are coded as A/B/C, A: unsuitable, without cavities, B: confounding, apparently suitable but without cavities, C: suitable, with cavities. For a description of the tree discrimination levels, see Methods section.

## Methods

To investigate the effect of tree discrimination, use of memory and information transfer on the effectiveness of the search for tree cavities, we simulated bats’ searching behaviour, using a spatially-explicit, individual based model. We first summarise the empirical background for the modelling framework used, as well as provide explanations for choices of parameter values used. Next, we present the description of the model itself.

We represent the process of roost finding and acquisition of information about the presence and distribution of roosts as follows (Figure 1): (1) The bat usually chooses the area to initiate the search for new cavities in the vicinity of known roosts. (2) It locates a potential roost tree using echolocation, vision, and knowledge about the spatial distribution of trees. Next, (3) the bat inspects the trunk surface from short distance (flying or crawling) in an attempt to detect an entrance to the cavity. If the search is unsuccessful the process of searching for new cavities continues from step 1 or 2. After finding the cavity (4) the bat inspects it and (5) evaluates its quality (6, 7). Information about the presence of a suitable new cavity is transferred to conspecifics through social calls that can be eavesdropped (8). Based on this information, the colony or part of the colony makes a decision on whether to use the cavity or not (9, 10). Once detected, the cavity enters the pool of suitable and known cavities which can be reused in the future.

### Insights into Searching for New Tree Cavities

*When to search?* Gleaning bats foraging in dense vegetation can potentially search for roosts while searching for prey (e.g. *Plecotus auritus*). Such behaviour would minimize additional energetic expenses. Species hunting in open space, far from trees, have to make an additional effort. New or known roosts are localized after the first foraging period, which indicates that bats are more willing to search for roosts when returning towards the original roost [53], [57].

*Where to search?* Bats usually select areas with a high density of trees suitable for roosting (e.g. [58], [59]). The mean distance between consecutive roosts is usually less than 400 m, which suggests that bats tend to use roosts in close vicinity to known roosts (see [17]). This decreases the costs of transporting juveniles, but also helps maintain group cohesion [53]; for review, see [17]).

*From what distance can bats detect a tree?* For bats using echolocation, the maximum range for detection of large objects does not exceed 90 m and is often limited to a few meters, particularly when obstacles are present [26], [27]. Visual cues allow for detection of objects from a further distance but this is limited to specific conditions [26], [60], [61]. Hence, we used two extreme values for the detection range: 5 m to represent a short detection range (e.g. in clutter environment) and 90 m as a long detection range (e.g. in open space).

**Figure 4:**
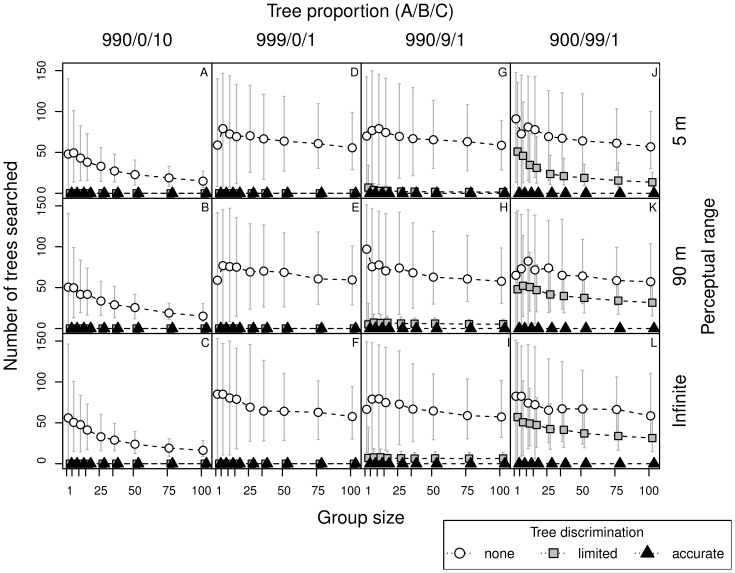
Number of trees inspected by bats, in relation to perceptual range, group size, tree discrimination skills and forest type. See caption to Figure 3 for details.

**Figure 5:**
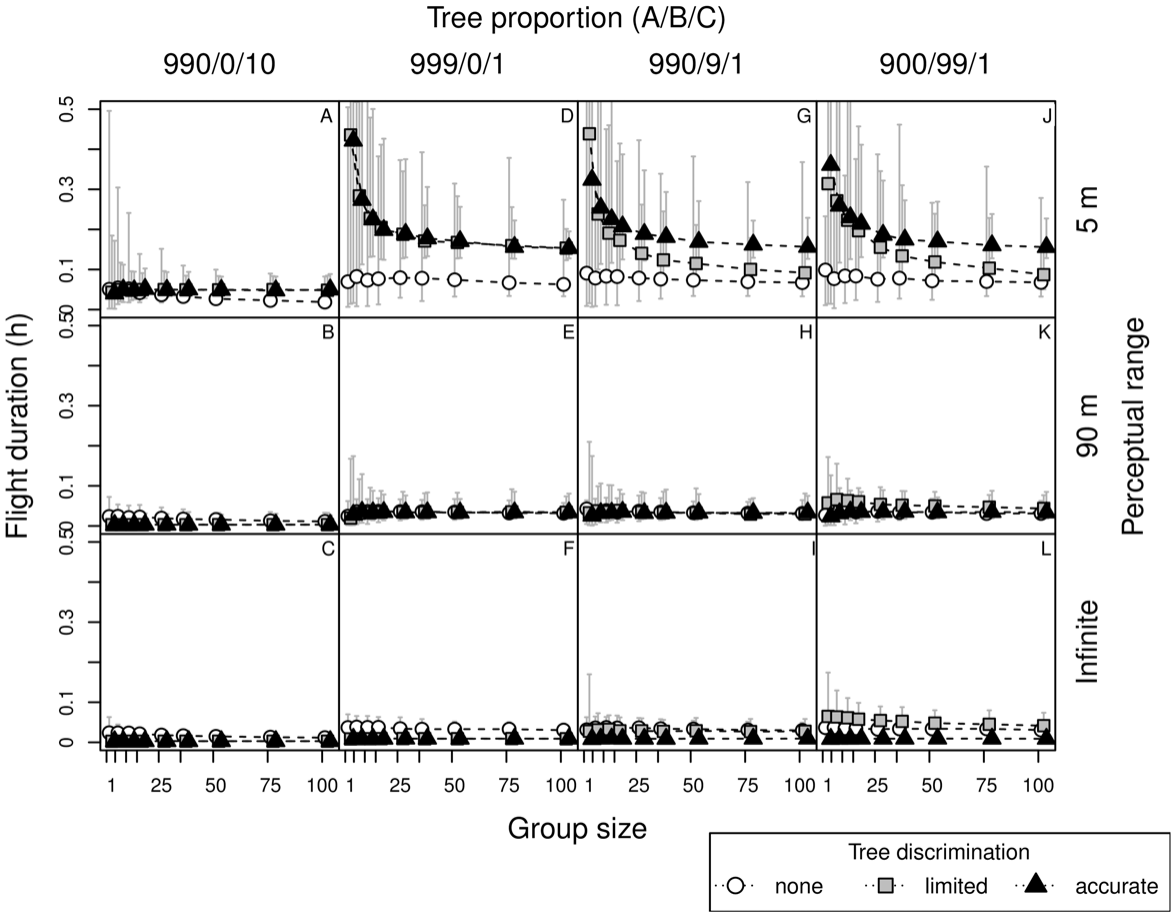
Flight duration in search for roosts, in relation to perceptual range, group size, tree discrimination skills and forest type. See caption to Figure 3 for details.

**Figure 6:**
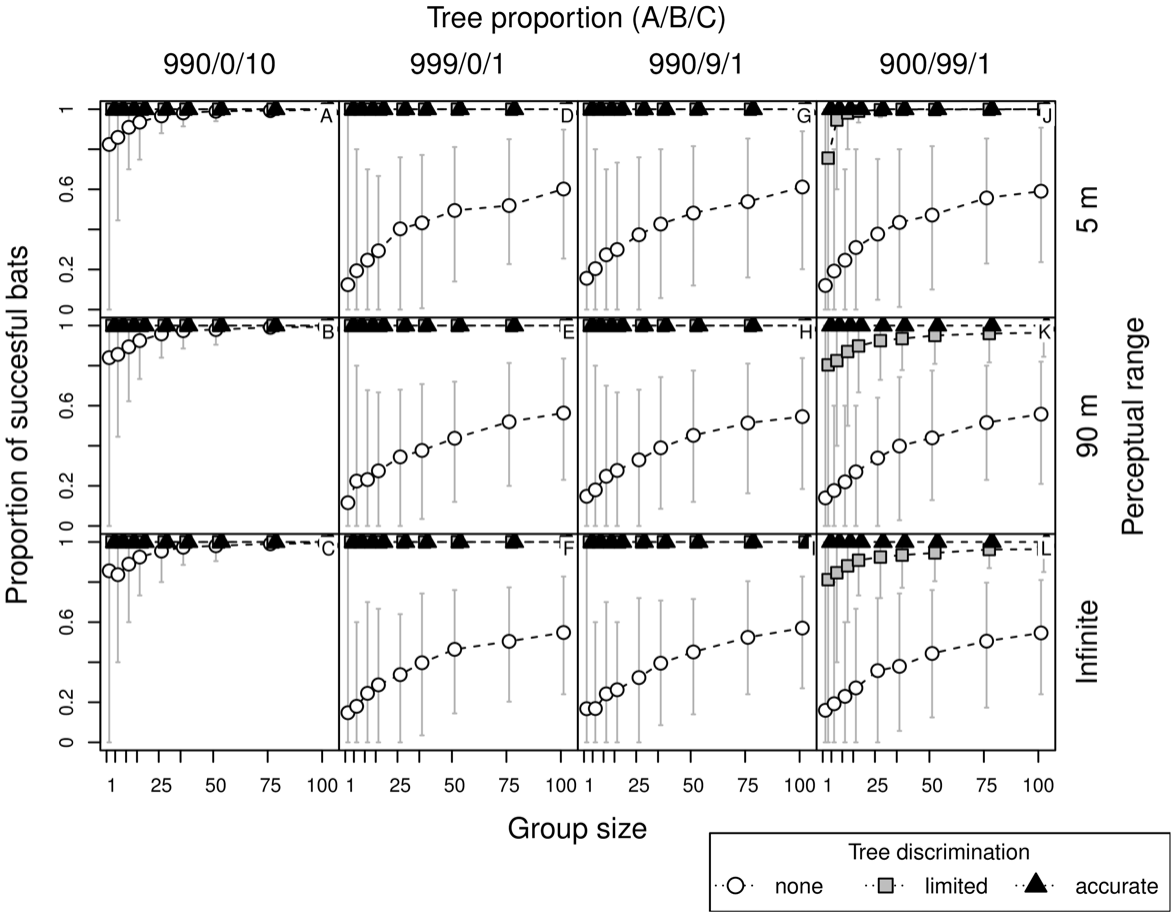
Proportion of successful bats in search for roosts, in relation to perceptual range, group size, tree discrimination skills and forest type. See caption to Figure 3 for details.

#### Distance of eavesdropping

Unlike echolocation calls, loud social calls, such as those produced by noctule bats *Nyctalus noctula* can be heard (eavesdropped) from a distance of at least 170 m [53]. Variation in the intensity of social calls among species does not allow for generalization of the eavesdropping range, so we used in the model a distance of 150 m. Apart from response to calls, we did not include any other types of interactions between individuals.

*How much time does a bat spend inspecting each tree?* Due to the difficulty of observing bats in the field, our knowledge of their searching strategies is extremely poor and limited to experiments conducted with artificial roosts such as bat boxes, or conducted under laboratory conditions [4], [34], [62]. Time needed to find a new tree cavity entrance is unknown. Trained bats under laboratory conditions needed around 45 s for finding the entrance in a 40 cm high artificial log [34]. Assuming that the search for new entrances under natural conditions is equally effective, and that bats check only the most promising part of the trunk (e.g. 5 m), we can estimate that bats need about 7.5 min for the inspection.

*Flight speed* varies among bat species and depends on factors such as environmental conditions and flight aims. Bats are quicker when commuting or migrating than during foraging [63]. Due to this large variation in flight speeds we arbitrarily chose 5 m/s for our model, which is between the maximum range speed and the minimum speed of *Myotis myotis* [64]. This speed is also within the range of many other bat species [63]–[65].

### The Model

We simulated bats searching for tree cavities using a spatially-explicit, individual based model. The model was implemented in C++ programming language, and the program is included in the Supporting Information (Model Code S1).

The forest was represented as trees randomly located in a continuous space (Figure 2). The average distance of 10 m between the trees represents a relatively sparse forest. Three types of trees were present in the forest: unsuitable, without cavities (type A), suitable with cavities (type C), and ‘confounding’, without cavities but perceptually similar to trees of type C (type B). The proportion of tree types varied between the simulations (Table 1).

Initially, the bats were displaced in a random direction within the searching range (*r_search_*) from the current roost tree (chosen at random, and common to all bats in the group). Subsequently, they started to search for another roost, with initial direction towards the current roost tree. The movement was done in a stepwise fashion, with a constant step length l, reflecting a speed of 5 m/s. The bat moved with a highly correlated random walk, following a nearly linear path. The correlated random walk was modelled by adding a uniform random noise to the turning angles in range *±θ*. If a tree fell within its perceptual range (*r_P_*), the bat began to move towards the tree in a straight line (left panel in Figure 2). To avoid the bats following deterministic trajectories, a small amount of random noise in the assessment of distance to the tree was introduced. We used (nearly) linear movement, as it has been found to be most efficient when searching for randomly distributed resources (e.g. [66]).

The searching behaviour was modelled as a simplified foray search, in which a bat reaching beyond a perimeter determined by the searching distance (*r_search_*) from the current roost tree would turn randomly with a certain per-step probability, unless there was a tree in sight (for an example, see left and middle panel of Figure 2). The individuals were not allowed to revisit the previously used roost tree (i.e. the one from which they started). Therefore, if they were unsuccessful in finding a suitable tree before reaching the previous roost tree, they changed direction at random and continued their flight. A tree was reached when the distance to the centre was lower than a step length (*l*). Following an encounter with a tree, a bat inspected the tree for the presence of a cavity, this activity takes *t_insp_*. If a cavity was found, the bat stayed in that tree, otherwise the bat memorized the visited tree and subsequently ignored it (Figure S1).

The simulation ended when all bats had found cavities or after 2^16^ time steps, whichever occurred first. For each individual, we recorded the number of inspected trees, *n_tree_*, the time spent flying between the inspections of trees (‘flight duration’, *t_flight_*), and, for successful bats, the time elapsed until a cavity was found, *t_search_* = *t_flight_*+*n_tree_ t_insp_*.

We used three levels of discriminatory skills, corresponding to the three tree types described above. Without tree discrimination, bats responded to presence of all types of trees. With ‘limited discrimination’, bats disregarded all trees of type A, investigating only the apparently suitable ones (type B or C). Finally, with ‘accurate discrimination’, bats ignored trees of both types A and B.

In addition to the short and long perceptual range (see previous section), we included another scenario, in which the bats possessed infinite perceptual range. Combined with accurate tree discrimination, this represents ‘memory’, which is in other words knowledge about distribution of all suitable trees. For consistency, we examined a full range of parameter combinations that also included less likely scenarios, for example those in which a bat had infinite perceptual range and limited or no tree discrimination ability.

In the next step we introduced information transfer between individuals (eavesdropping of social calls), and simulated release of a group of bats (*n* = 5, 10, 75, 100 individuals). Initially, the search proceeded as described above. After discovering a tree cavity and settling in at that cavity, the individual started calling, which extended the detection distance of that tree to *r_call_ = *150 m. When other bats were present within the calling range, they directed their flight towards that tree. The calling continued until the end of the simulation. The flight mode could be therefore either a search in order to detect a tree, or a directed flight towards the detected target tree. When flying directly, the bat would not switch over to another target, i.e. when another bat started calling within a distance of *r_call_*. A detailed procedure is presented in the Supporting Information (Figure S1).

We ran simulations with all combinations of different proportions of tree types, three perceptual ranges (low, high and infinite), three levels of discriminatory skills (none, limited and accurate), with individual bats as well as with groups of varying sizes (the latter involving social calls). Table 1 summarizes all the parameter values used. For each parameter combination we replicated the simulation 250 times.

## Results

### Perceptual Range and Memory

Bats with short (5 m) and long (90 m) perceptual range needed a similar period of time to find tree cavities, as those possessing knowledge about the distribution of trees in the landscape (i.e. infinite perceptual range; compare search time of bats with the same discrimination abilities, Figure 3). However, bats accurately recognizing trees, but with short perceptual range, needed more time than those with long-range perception in forests with a low density of suitable trees (Table 2). In our model the bats always move towards the closest recognized tree (depending on the discrimination level), therefore there was no advantage of perceptual range when it exceeded the average distance between the recognized trees (compare panels for large and infinite perceptive range in Figure 3).

### Tree Discrimination

Bats without discrimination ability searched for more than 1.5 h, even in forests with a high proportion of suitable trees (1/100; 1.8–6.5 h). When the proportion of trees with cavities was one magnitude lower (1/1000), search time increased several times (in Figure 3 compare A–C with D–L). With a high proportion of suitable trees (1/100), bats accurately discriminating trees needed less than 5 min to find a cavity (Figure 3 A–C). In forests with a small proportion of suitable trees with cavities (1/1000), time to find a cavity was still shorter than 0.5 h (Figure 3 D–L).

Under natural conditions the tree discrimination performance likely falls between ‘none’ and ‘accurate’. We compared effectiveness of finding new tree cavities by bats with limited ability for tree discrimination (i.e. responding only to trees of type B and C). Obviously, when there are no trees of type B, performance of bats with this limited discrimination is identical to that of bats recognizing trees accurately (Figure 3 D–F). As the proportion of confounding trees of type B increases (in forest with tree proportion ‘990/9/1′) the search time also increases (Figure 3 G–I and J–L).

As bats with limited discrimination ignore trees of type A, the effective proportion of suitable trees in the forest ‘900/99/1′ was the same as in the forest ‘990/0/10′ for non-discriminating bats (1/100). Thus the results in these two cases were similar (Figure 3 A–C versus Figure 3 J–L). The difference, however, is in the density of recognized trees, or more precisely in the relative scale of between-tree distances to perceptual range (respectively 1 and 5 units, with perceptual range being 0.5 and 9 units). The search time and number of trees visited (Figures 3 and 4) are comparable, yet flight durations are longer (Figure 5 A–C versus Figure 5 J–L). As the flight duration was negligible compared to the time needed for inspection of each tree, the overall search time was primarily influenced by the number of inspected trees.

### Information Transfer - Eavesdropping

As the group size increased, the positive effect of eavesdropping on searching success became more pronounced (Figures 3, 4, 5). Eavesdropping was primarily important for non-discriminating bats, in which the proportion of successful bats increased asymptotically (Figure 6) with group size. Search time decreased with the number of bats in a colony, also in forests with a high proportion of confounding trees (type B, Figure 3 J–L). Large perceptual range only slightly improved performance in larger groups with limited discrimination ability. This arises, because while searching (when there are no trees within perception range) bats were more responsive to the informing calls of other bats, as they are less preoccupied by inspecting the trees (in the model bats were not allowed to interrupt that activity). Eavesdropping played no or only a minor role when animals were able to recognize trees with cavities accurately (Figure 3 A–L).

## Discussion

We compared potential benefits of strategies of compensating (tree discrimination, memory and information transfer) for limited perceptual range in searches for tree cavities, by low and high tree cavity availability. The results from modelling suggest that: 1) perceptual range may play a minor role in finding new roosts; 2) correct remote classification of tree quality (tree discrimination) is crucial for roost finding, chiefly when the probability of encountering an unsuitable tree is high; 3) use of memory decreases search time; 4) transferring information through eavesdropping decreases the search time when the tree discrimination skills of bats are low, and risk of error is high; and 5) a larger number of bats increases effectiveness of eavesdropping.

### Perceptual Range, Memory and Tree Discrimination

Short echolocation range is common in many forest dwelling bat species [26], [27], [67], [68]. Animals using echolocation usually forage close to vegetation and are not able to detect objects from a longer distance. In our results, the differences in search time between bats with short, long and infinite perceptual range were nonetheless very small. Flight allows for fast movement between inspected trees and the proportion of time spent in travel had limited influence on the total search time. Longer perceptual range is unlikely to confer obvious benefits if search costs are related mainly to the process of tree inspection. Consequently, our results suggest that searching effectiveness (regardless of tree discrimination level, information transfer, and density of tree cavities) may be little dependent on bat perceptual range.

It must be noted that our results as to the effect of perceptual range are influenced by the movement strategy considered. Since the individuals in our model respond to the closest tree within their perceptual range, and the trees are randomly distributed, the effect of perceptual range is limited to the average distance to recognized trees. Yet, movement in the direction of the closest target within a detection range seems to be a likely behaviour.

In forest with low density of suitable trees, we nonetheless observed an increase in the duration of flight in the total search time, when the animals had short perceptual range. This indicates that while the benefits of directional flight exist, they are relatively low. Moreover, the benefits are related to the distance between inspected trees; lower tree density increases the proportion of time spent flying during the search. Bats are very mobile, which makes such a scenario plausible owing to seasonal habitat changes. For instance, during migration, when bats travel long distances to new habitats inspecting only a small number of the most promising trees, the distance between inspected trees is long and so is the time spent in flight.

In almost all considered scenarios, the ability for tree discrimination was crucial for decreasing costs of finding new cavities, mainly because it minimized the number of inspected trees. Advantages of correct classification of trees may include decreased search time and, consequently, lower energetic costs of flight [69], as well as reduced predation risk [70], [71]. This compensation strategy offers important benefits, especially if there is a high cost of examining unsuitable trees. Identification of the most promising trees by bats requires appropriate echolocation or visual markers, similar to the acoustic guides present in some bat pollinated flowers [72], that can be associated with the presence of numerous and high quality cavities, such as large old trees and snags [40], [73]–[75]. Finding cavities in very uniform habitats, such as forest plantations, where trees with and without cavities appear very similar would be much more demanding and time consuming, because ineffective tree discrimination, as observed in scenario with large proportion of confounding trees, immediately increase search time. Our current results point out implications for forest management policies, which should aim to preserve older or dead trees (such as snags) that offer echolocatory or visual guides which can be associated with the presence of high quality cavities. Bats can also decrease searching costs by inspecting more obvious objects such as artificial boxes. Advantages for investment in cognitive skills allowing better discrimination of trees may be particularly high when combined with selection of habitats that offer higher chances of a correct choice.

### Information Transfer

In many social animals, such as insects or birds, public information can decrease costs of finding new resources (for review, see [76], [77]). It is known that the interaction between food distribution and exploratory behaviour affect the development of social structure e.g. in crabs [2]. The role of information transfer for finding roosts by bats has recently been studied intensively [13], [53], [56]. To our knowledge, this model presents the first evaluation of potential benefits associated with formation of larger groups for finding new roosts. Group size may play an important role in decreasing the costs of finding roosts when bats are unable to recognize the suitable trees effectively on their own, especially when there is low proportion of such trees. Individual bats which did not classify trees correctly required an extended time period in order to find a cavity, whereas groups of bats in the same environment and with the same cognitive skills may find new cavities within a considerably shorter time.

In contrast, the role of group size in decreasing search time was marginal when individuals were capable of effective tree identification. This indicates that eavesdropping is more important in situations where tree discrimination fails, for example due to habitat conditions. Hence, natural selection should promote larger groups when resource identification abilities of individuals cannot be improved, but not when cognitive skills and environmental conditions allow for rapid detection of these resources. Accordingly, cognitive skills necessary for finding tree cavities (associative skills and memory) may indirectly shape colony size. Although there are additional reasons for group living [18],[51], our model illustrates the links between the environment (proportion of suitable trees), cognitive skills and the colony size of bats. In our model we did not consider other strategies of information transfer, such as following behaviour, which could further increase the effectiveness of roost finding [13], [52], [78], [79]. Nevertheless, even with our conservative approach utilising the simplest mechanism for information transfer, the results show advantages of living in groups under specific circumstances.

### Search Time

It is unclear how much time bats need for finding new tree cavities under natural conditions. Several species, including *Chalinolobus tuberculatus* [39], use new trees almost every day and thus need to find new cavities very efficiently. Our simulations indicate that bats may be able to find a new cavity within a short time, but only if either identification of trees is accurate, or chances for misidentification are small. In most scenarios without tree discrimination, individual bats would spend up to 15 hours finding a new cavity. Females of *Myotis bechsteinii* need 1–2 weeks to find new artificial boxes [24]. However, bats may take several months or years to start using tree boxes (e.g. [80], [81]. This may indicate the difficulties of finding new boxes and also the lack of pressure to find new roosts. In our model, bats were able to find a cavity within 4–10 days if they spent around 30 min searching every night in a habitat with a low density of suitable tree cavities. Therefore, natural selection is likely to act on strategies that decrease roost detection costs.

Generally, all considered potential strategies of compensating for sensory limitation may provide advantage with search for tree cavities. While efficiency of social calls depends on the group size, the value of individual ability to remotely discriminate unsuitable resources and memorize their locations depends mainly on the habitat composition and quality of the resources. The simple strategic model presented here highlights the complexity of relationships between sensory limitation, cognition, sociality and environment, and empirical verification of our results would be an interesting task for further studies.

## Supporting Information

Figure S1 Flowchart representing the procedure for finding new tree cavities, as implemented in the simulation program.
(PDF)

Model Code S1 C++ code and Windows binary of the simulation program.
(ZIP)

## References

[1] Kerth G, Reckardt K (2003) Information transfer about roosts in female Bechstein’s bats: an experimental field study. Proceedings of the Royal Society of London Series B-Biological Sciences 270: 511–515. 10.1098/rspb.2002.2267 PMC1691266 12641906

[2] Tanner CJ, Jackson AL (2012) Social structure emerges via the interaction between local ecology and individual behaviour. Journal of Animal Ecology 81: 260–267. 21668891 10.1111/j.1365-2656.2011.01879.x

[3] Nevitt G (1999) Olfactory foraging in Antarctic seabirds: a species-specific attraction to krill odors. Marine Ecology-Progress Series 177: 235–241.

[4] Ruczyński I, Kalko EKV, Siemers BM (2007) The sensory basis of roost finding in a forest bat, *Nyctalus noctula* . Journal of Experimental Biology 210: 3607–3615. 17921162 10.1242/jeb.009837

[5] Niven JE, Laughlin SB (2008) Energy limitation as a selective pressure on the evolution of sensory systems. Journal of Experimental Biology 211: 1792–1804. 18490395 10.1242/jeb.017574

[6] Rettie WJ, Messier F (2000) Hierarchical habitat selection by woodland caribou: its relationship to limiting factors. Ecography 23: 466–478.

[7] Dittman AH, Quinn TP (1996) Homing in Pacific salmon: Mechanisms and ecological basis. Journal of Experimental Biology 199: 83–91. 9317381 10.1242/jeb.199.1.83

[8] Emery NJ (2006) Cognitive ornithology: the evolution of avian intelligence. Philosophical Transactions of the Royal Society B-Biological Sciences 361: 23–43. 10.1098/rstb.2005.1736 PMC1626540 16553307

[9] Danchin E, Giraldeau LA, Valone TJ, Wagner RH (2004) Public information: From nosy neighbors to cultural evolution. Science 305: 487–491. 15273386 10.1126/science.1098254

[10] Dall SRX, Giraldeau LA, Olsson O, McNamara JM, Stephens DW (2005) Information and its use by animals in evolutionary ecology. Trends in Ecology & Evolution 20: 187–193. 16701367 10.1016/j.tree.2005.01.010

[11] Lučan RK, Hanák V, Horáček I (2009) Long-term re-use of tree roosts by European forest bats. Forest Ecology and Management 258: 1301–1306.

[12] Lewis SE (1995) Roost Fidelity of Bats - a Review. Journal of Mammalogy 76: 481–496.

[13] Chaverri G, Gillam EH, Vonhof MJ (2010) Social calls used by a leaf-roosting bat to signal location. Biology Letters 6: 441–444. 20071395 10.1098/rsbl.2009.0964 PMC2936192

[14] Gaudet CL, Fenton MB (1984) Observational-learning in 3 species of insectivorous bats (Chiroptera). Animal Behaviour 32: 385–388.

[15] Siemers BM, Schnitzler HU (2004) Echolocation signals reflect niche differentiation in five sympatric congeneric bat species. Nature 429: 657–661. 15190352 10.1038/nature02547

[16] Dechmann DKN, Heucke SL, Giuggioli L, Safi K, Voigt CC, et al (2009) Experimental evidence for group hunting via eavesdropping in echolocating bats. Proc R Soc Lond B Biol Sci 276: 2721–2728. 10.1098/rspb.2009.0473 PMC2839959 19419986

[17] Kunz TH, Lumsden LF (2003) Ecology of cavity and foliage roosting bats. Chicago: University of Chicago Press. 3–89 p.

[18] Willis CKR, Brigham RM (2007) Social thermoregulation exerts more influence than microclimate on forest roost preferences by a cavity-dwelling bat. Behavioral Ecology and Sociobiology 62: 97–108.

[19] Ruczyński I (2006) Influence of temperature on maternity roost selection by noctule bats (*Nyctalus noctula*) and Leisler’s bats (*N. leisleri*) in Białowieża Primeval Forest, Poland. Canadian Journal of Zoology-Revue Canadienne De Zoologie 84: 900–907.

[20] Sedgeley JA (2001) Quality of cavity microclimate as a factor influencing selection of maternity roosts by a tree-dwelling bat, *Chalinolobus tuberculatus*, in New Zealand. Journal of Applied Ecology 38: 425–438.

[21] Reiter G (2004) The importance of woodland for Rhinolophus hipposideros (Chiroptera, Rhinolophidae) in Austria. Mammalia 68: 403–410.

[22] Zahn A (1999) Reproductive success, colony size and roost temperature in attic-dwelling bat *Myotis myotis* . Journal of Zoology 247: 275–280.

[23] Sedgeley JA, O’Donnell CFJ (1999) Factors influencing the selection of roost cavities by a temperate rainforest bat (Vespertilionidae : Chalinolobus tuberculatus) in New Zealand. Journal of Zoology 249: 437–446.

[24] Kerth G, Ebert C, Schmidtke C (2006) Group decision making in fission-fusion societies: evidence from two-field experiments in Bechstein’s bats. Proceedings of the Royal Society B-Biological Sciences 273: 2785–2790. 10.1098/rspb.2006.3647 PMC1635504 17015328

[25] Kerth G, Perony N, Schweitzer F (2011) Bats are able to maintain long-term social relationships despite the high fission-fusion dynamics of their groups. Proceedings of the Royal Society B-Biological Sciences 278: 2761–2767. 10.1098/rspb.2010.2718 PMC3145188 21307051

[26] Schnitzler HU, Kalko EKV (2001) Echolocation by insect-eating bats. Bioscience 51: 557–569.

[27] Holderied MW, von Helversen O (2003) Echolocation range and wingbeat period match in aerial-hawking bats. Proceedings of the Royal Society of London Series B 270: 2293–2299. 14613617 10.1098/rspb.2003.2487 PMC1691500

[28] Holland RA (2007) Orientation and navigation in bats: known unknowns or unknown unknowns? Behavioral Ecology and Sociobiology 61: 653–660.

[29] Tsoar A, Nathan R, Bartan Y, Vyssotski A, Dell’Omo G, et al (2011) Large-scale navigational map in a mammal. Proceedings of the National Academy of Sciences of the United States of America 108: E718–E724. 21844350 10.1073/pnas.1107365108 PMC3174628

[30] Eklöf J, Jones G (2003) Use of vision in prey detection by brown long-eared bats, *Plecotus auritus* . Animal Behaviour 66: 949–953.

[31] Eklöf J, Svensson AM, Rydell J (2002) Northern bats, *Eptesicus nilssonii*, use vision but not flutter-detection when searching for prey in clutter. Oikos 99: 347–351.

[32] Winter Y, Lopez J, von Helversen O (2003) Ultraviolet vision in a bat. Nature 425: 612–614. 14534585 10.1038/nature01971

[33] Rydell J, Eklöf J (2003) Vision complements echolocation in an aerial-hawking bat. Naturwissenschaften 90: 481–483. 14564410 10.1007/s00114-003-0464-x

[34] Ruczyński I, Kalko EKV, Siemers BM (2009) Calls in the forest: how bats find tree cavities. Ethology 115: 167–177.

[35] Ruczyński I SA, Siemers BM (2011) Conspicuous visual cues can help bats to find tree cavities. Acta Chiropterologica 13: 385–389.

[36] Koch AJ, Munks SA, Driscoll D, Kirkpatrick JB (2008) Does hollow occurrence vary with forest type? A case study in wet and dry Eucalyptus obliqua forest. Forest Ecology and Management 255: 3938–3951.

[37] Fox JC, Hamilton F, Occhipinti S (2009) Tree hollow incidence in Victorian state forests. Australian Forestry 72: 39–48.

[38] Ojeda VS, Suarez ML, Kitzberger T (2007) Crown dieback events as key processes creating cavity habitat for magellanic woodpeckers. Austral Ecology 32: 436–445.

[39] Sedgeley JA, O’Donnell CFJ (1999) Roost selection by the long-tailed bat, *Chalinolobus tuberculatus*, in temperate New Zealand rainforest and its implications for the conservation of bats in managed forests. Biological Conservation 88: 261–276.

[40] Grunwald JE, Schörnich S, Wiegrebe L (2004) Classification of natural textures in echolocation. Proceedings of the National Academy of Sciences of the United States of America 101: 5670–5674. 15060282 10.1073/pnas.0308029101 PMC397469

[41] Siemers BM (2001) Finding prey by associative learning in gleaning bats: experiments with a Natterer’s bat *Myotis nattereri* . Acta Chiropterologica 3: 211–215.

[42] Siemers BM, Schnitzler HU (2000) Natterer’s bat (*Myotis nattereri* Kuhl, 1818) hawks for prey close to vegetation using echolocation signals of very broad bandwidth. Behavioral Ecology and Sociobiology 47: 400–412.

[43] Page RA, Ryan MJ (2005) Flexibility in assessment of prey cues: frog-eating bats and frog calls. Proceedings of the Royal Society B-Biological Sciences 272: 841–847. 10.1098/rspb.2004.2998 PMC1599868 15888417

[44] von Helversen D (2004) Object classification by echolocation in nectar feeding bats: size-independent generalization of shape. Journal of Comparative Physiology a-Neuroethology Sensory Neural and Behavioral Physiology 190: 515–521. 10.1007/s00359-004-0492-9 15103497

[45] Yovel Y, Franz MO, Stilz P, Schnitzler H–U (2008) Plant classification from bat-like echolocation signals. Plos Computational Biology 4. 10.1371/journal.pcbi.1000032 PMC2267002 18369425

[46] Yovel Y, Franz MO, Stilz P, Schnitzler H–U (2011) Complex echo classification by echo-locating bats: a review. Journal of Comparative Physiology a-Neuroethology Sensory Neural and Behavioral Physiology 197: 475–490. 10.1007/s00359-010-0584-7 20848111

[47] Yovel Y, Stilz P, Franz MO, Boonman A, Schnitzler H–U (2009) What a Plant Sounds Like: The Statistics of Vegetation Echoes as Received by Echolocating Bats. Plos Computational Biology 5. 10.1371/journal.pcbi.1000429 PMC2699101 19578430

[48] Wilkinson GS, South JM (2002) Life history, ecology and longevity in bats. Aging Cell 1: 124–131. 12882342 10.1046/j.1474-9728.2002.00020.x

[49] Willis CKR, Kolar KA, Karst AL, Kalcounis-Rueppell MC, Brigham RM (2003) Medium- and long-term reuse of trembling aspen cavities as roosts by big brown bats (*Eptesicus fuscus*). Acta Chiropterologica 5: 85–90.

[50] Krause J, Ruxton GD (2002) Living in Groups. Oxford: Oxford University Press.

[51] Safi K, Kerth G (2007) Natural history miscellany - Comparative analyses suggest that information transfer promoted sociality in male bats in the temperate zone. American Naturalist 170: 465–472. 10.1086/520116 17879196

[52] Wilkinson GS (1992) Information transfer at evening bat colonies. Animal Behaviour 44: 501–518.

[53] Furmankiewicz J, Ruczyński I, Urban R, Jones G (2011) Social Calls Provide Tree-dwelling Bats with Information about the Location of Conspecifics at Roosts. Ethology 117: 480–489.

[54] Kaňuch P (2007) Evening and morning activity schedules of the noctule bat (*Nyctalus noctula*) in Western Carpathians. Mammalia 71: 126–130.

[55] Laland KN (2004) Social learning strategies. Learning & Behavior 32: 4–14. 15161136 10.3758/bf03196002

[56] Schöner CR, Schöner MG, Kerth G (2010) Similar is not the same: Social calls of conspecifics are more effective in attracting wild bats to day roosts than those of other bat species. Behavioral Ecology and Sociobiology 64: 2053–2063.

[57] Vonhof MJ, Betts BJ (2010) Nocturnal activity patterns of lactating silver-haired bats (*Lasionycteris noctivagans*): the influence of roost-switching behavior. Acta Chiropterologica 12: 283–291.

[58] Russo D, Cistrone L, Jones G, Mazzoleni S (2004) Roost selection by barbastelle bats (*Barbastella barbastellus*, Chiroptera : Vespertilionidae) in beech woodlands of central Italy: consequences for conservation. Biological Conservation 117: 73–81.

[59] Ruczyński I, Nicholls B, MacLeod CD, Racey PA (2010) Selection of roosting habitats by *Nyctalus noctula* and *Nyctalus leisleri* in Białowieża Forest-Adaptive response to forest management? Forest Ecology and Management 259: 1633–1641.

[60] Eklöf J (2003) Vision in echolocating bats [PhD]. Göteborg: Göteborg University. 477 p.

[61] Suthers RA, Wallis NE (1970) Optics of eyes of echolocating bats. Vision Research 10: 1165–1173. 5508963 10.1016/0042-6989(70)90034-9

[62] Kerth G (2006) Group decision-making in fission-fusion societies. Behavioural Processes 84: 662–663. 10.1016/j.beproc.2010.02.023 20211711

[63] Hedenstrom A (2009) Optimal Migration Strategies in Bats. Journal of Mammalogy 90: 1298–1309.

[64] Britton ARC, Jones G, Rayner JMV, Boonman AM, Verboom B (1997) Flight performance, echolocation and foraging behaviour in pond bats, *Myotis dasycneme* (Chiroptera: Vespertilionidae). Journal of Zoology 241: 503–522.

[65] Winter Y (1999) Flight speed and body mass of nectar-feeding bats (Glossophaginae) during foraging. Journal of Experimental Biology 202: 1917–1930. 10377273 10.1242/jeb.202.14.1917

[66] Zollner PA, Lima SL (1999) Search strategies for landscape-level interpatch movements. Ecology 80: 1019–1030.

[67] Jones G (1999) Scaling of echolocation call parameters in bats. Journal of Experimental Biology 202: 3359–3367. 10562518 10.1242/jeb.202.23.3359

[68] Barclay RMR, Brigham RM (1991) Prey detection, dietary niche breadth, and body size in bats - Why are aerial insectivorous bats so small. American Naturalist 137: 693–703.

[69] Voigt CC, Winter Y (1999) Energetic cost of hovering flight in nectar-feeding bats (Phyllostomidae: Glossophaginae) and its scaling in moths, birds and bats. Journal of Comparative Physiology B-Biochemical Systemic and Environmental Physiology 169: 38–48. 10.1007/s003600050191 10093905

[70] Speakman JR (1991) The Impact of predation by birds on bat populations in the British-Isles. Mammal Review 21: 123–142.

[71] Czeszczewik D, Walankiewicz W, Stańska M (2008) Small mammals in nests of cavity-nesting birds: Why should ornithologists study rodents? Canadian Journal of Zoology-Revue Canadienne De Zoologie 86: 286–293.

[72] von Helversen D, von Helversen O (2003) Object recognition by echolocation: a nectar-feeding bat exploiting the flowers of a rain forest vine. Journal of Comparative Physiology a-Neuroethology Sensory Neural and Behavioral Physiology 189: 327–336. 10.1007/s00359-003-0405-3 12712362

[73] Ruczyński I, Bogdanowicz W (2008) Summer roost selection by tree-dwelling bats *Nyctalus noctula* and *N. leisleri*: A multiscale analysis. Journal of Mammalogy 89: 942–951.

[74] Kalcounis-Ruppell MC, Psyllakis JM, Brigham RM (2005) Tree roost selection by bats: an empirical synthesis using meta-analysis. Wildlife Society Bulletin 33: 1123–1132.

[75] Ruczyński I, Bogdanowicz W (2005) Roost cavity selection by *Nyctalus noctula* and *N. leisleri* (Vespertilionidae, Chiroptera) in Białowieża Primeval Forest, eastern Poland. Journal of Mammalogy 86: 921–930.

[76] Ward P, Zahavi A (1973) Inportance of certain assemblages of birds as information-centers for food-feeding. Ibis 115: 517–534.

[77] Chittka L, Leadbeater E (2005) Social learning: Public information in insects. Current Biology 15: R869–R871. 16271856 10.1016/j.cub.2005.10.018

[78] Spanjer Wright G, Wilkinson GS, Moss CF (2011) Social learning of a novel foraging task by big brown bats, Eptesicus fuscus. Animal Behaviour 82: 1075–1083. 22328786 10.1016/j.anbehav.2011.07.044 PMC3274777

[79] Gillam EH, O’Shea TJ, Brigham RM (2011) Nonrandom patterns of roost emergence in big brown bats, Eptesicus fuscus. Journal of Mammalogy 92: 1253–1260.

[80] Ciechanowski M (2005) Utilization of artificial shelters by bats (Chiroptera) in three different types of forest. Folia Zoologica 54: 31–37.

[81] Krzanowski A (1961) Wyniki rozwieszenia skrzynek dla nietoperzy w Białowieskim Parku Narodowym. Chrońmy Przyrodę Ojczystą 17: 29–32.

