# Supplementary figures and images for: Modelling Sensory Limitation: The Role of Tree Selection, Memory and Information Transfer in Bats’ Roost Searching Strategies
Source: PLoS One. 2012 Sep 13;7(9):e44897. doi: 10.1371/journal.pone.0044897 (PMC3441568; doi:10.1371/journal.pone.0044897)

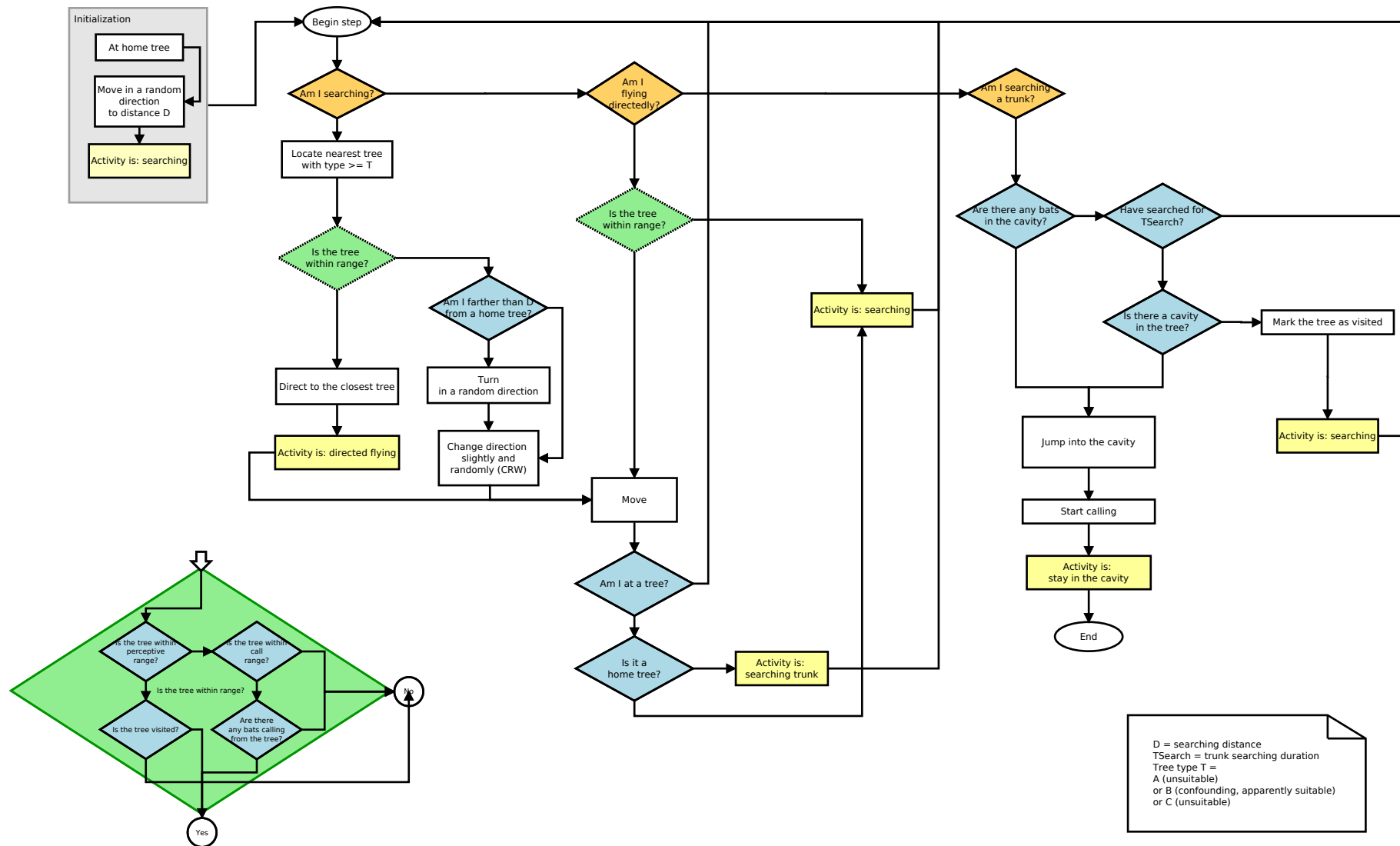

Supplement: Figure S1 — Flowchart representing the procedure for finding new tree cavities, as implemented in the simulation program. (PDF) [file pone.0044897.s001.pdf]
